# Pyelolithotomy in a Patient with Complete Coraliform Calcification Induced by a Double-J Ureteral Stent

**DOI:** 10.1155/2019/6957141

**Published:** 2019-05-28

**Authors:** Miguel Bonfitto, Analaura de Oliveira Cury, Victoria Caroline Pagelkopf, Vinicius Ramos Bezerra de Morais, Carlos Abib Cury

**Affiliations:** ^1^São José do Rio Preto Base Hospital/Famerp, SP, Brazil; ^2^Votuporanga School of Medicine, SP, Brazil; ^3^São José do Rio Preto School of Medicine (Famerp), SP, Brazil; ^4^Urology Sector, São José do Rio Preto School of Medicine (Famerp), SP, Brazil

## Abstract

The placement of a double-J ureteral stent enables the treatment of upper urinary tract obstruction. Despite advances, forgetting the stent favors the occurrence of calcification, leading to increased morbidity rates, lawsuits, and a financial burden on the healthcare system. This paper describes a successful pyelolithotomy for the removal of a calcified double-J ureteral stent.

## 1. Introduction

The placement of a double-J ureteral stent enables the draining and relief of obstructive processes of the upper urinary tract [1]. Despite the benefits of this stent and its innovations, complications stemming from the implanted stent constitute an important cause of morbidity [1]. The main complications are occlusion, calcification, migration, fragmentation, the formation of calculus, and compromised kidney function, which can be caused by oversaturation or the long-term presence of the stent [1, 2]. The removal of a calcified stent can be a challenge to urologists and requires procedures ranging from noninvasive extracorporeal lithotripsy to open surgery [1–4].

We report the difficult treatment of a patient with complete coraliform calcification of a double-J stent, highlighting the importance of avoiding complications secondary to the presence of the ureteral stent, as such factors contribute to patient morbidity and mortality.

## 2. Presentation of Case

**Figure 1:**
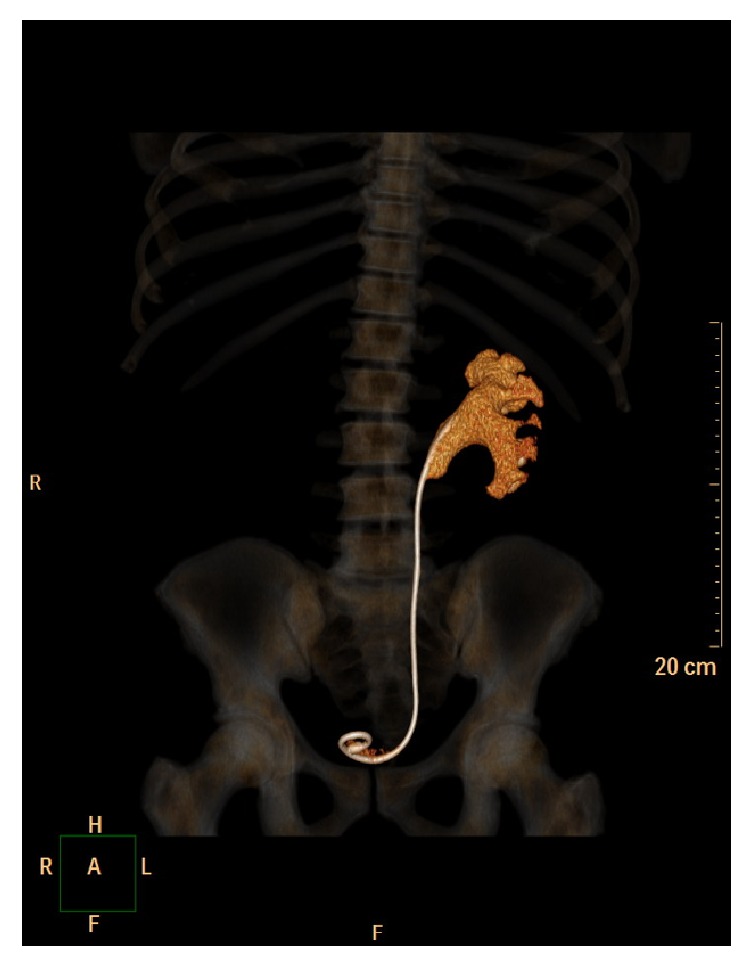
Abdominal computed tomography (coronal cut) showing complete coraliform calcification on double-J stent.

**Figure 2:**
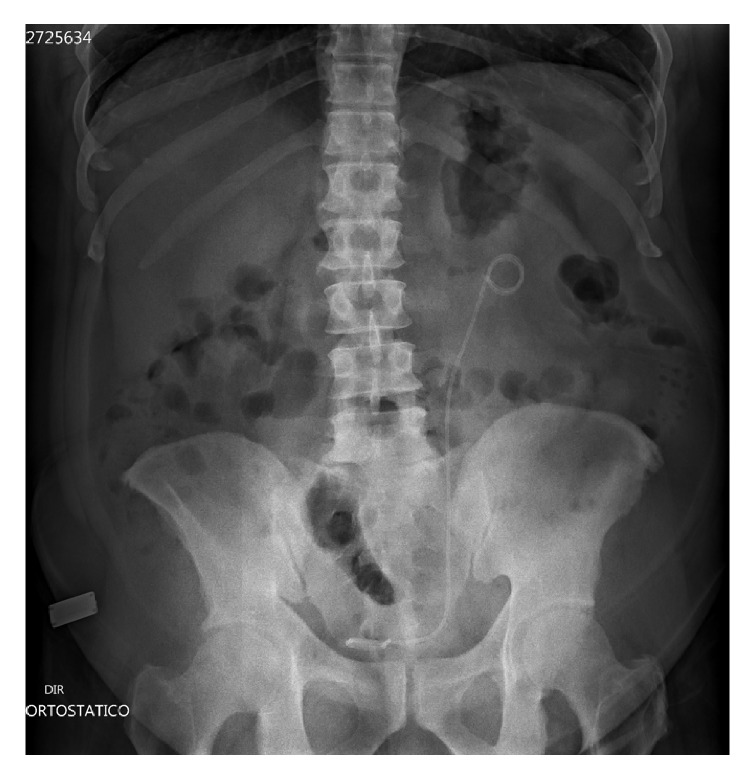
Abdominal X-ray showing absence of residual calculi in left pyelocalicial system.

A 38-year-old male patient was sent to the urology clinic due to difficulty in removing a calcified double-J stent in the left ureter. The patient reported that he had sought a different medical center seven months earlier with the complaint of left-side renal colic associated with nausea and vomiting and was diagnosed with a ureteral stone. After referral to our service, the patient was asymptomatic and the physical examination revealed a good general health status with a flat, painless abdomen. Cystoscopy performed for the removal of the stent was unsuccessful. The patient was then submitted to computed tomography of the kidneys and urinary ducts (Figure 1), which revealed a stent on the left side with complete coraliform calcification extending to the ureteropelvic junction and obliteration of the adjacent fat. The patient was submitted to extracorporeal lithotripsy without success. Thirteen months after admission, extended pyelolithotomy was performed with complete dissection of the renal sinus, exposing the entire pelvis, followed by a U-shaped incision around the renal sinus, enabling access to all renal infundibula. The calcified double-J stent was then removed from the renal sinus toward the ureteropelvic junction. The patient demonstrated good postoperative evolution, with the absence of residual calculi in the follow-up abdominal X-ray (Figure 2), receiving discharge on the third day. The patient is currently in follow-up.

## 3. Step by Step

1. Left lumbar incision and opening of planes (superficial, muscular, pararenal, and perirenal), enabling retroperitoneal access to the kidney.
2. Isolation of proximal ureter, with ascending dissection of the renal pelvis and left renal sinus, with ample exposure of the field to be operated.
3. Inverted U-shaped incision in posterior portion of renal pelvis at its transition with the calyx infundibula, obtaining access to the double-J catheter completed encompassed by the pelvic calcified mass and its calyx branches.
4. Anterograde removal of calcified double-J catheter from the renal sinus to the ureteropelvic junction.

## 4. Discussion

The double-J stent is a therapeutic option for different urological conditions [5, 6]. The main risk factors for the calcification of this stent are low schooling, time of use, sepsis, pyelonephritis, chronic kidney disease, recurring or residual kidney stones, congenital and metabolic abnormalities, and malignant ureteral obstruction due to chemotherapy with hyperuricosuria [5]. Physiological changes during pregnancy can also predispose the patient to calcification of the stent [6]. When removal by cystoscopy is not possible due to the calcification, another procedure is required [5, 6].

Treatment is generally performed using endourological (transurethral) procedures, with the rare need for open surgical techniques for patients in whom the stent remains for up to 30 months [7]. In such cases, percutaneous nephrolithotomy achieves good results. However, patients treated with this procedure for the resolution of complete calculi have residual calculi in up to 70% of times, which increases the risk of obstructive conditions in the postoperative period [8, 9]. The possible approaches to the removal of a calcified double-J stent are cystoscopy, extracorporeal lithotripsy, cystolithotripsy, ureterolithotripsy, percutaneous nephrolithotomy, or open surgery [9–13]. The video-assisted laparoscopic and robotic approaches can be indicated in select cases as methods for the treatment of coraliform calculi and pelvic calculi of a medium size and favorable anatomic access (extrarenal pelvis) [14–18]. Regarding the double-J catheter calcified in complete coraliform calculus, there is no report in the indexed literature on such techniques to date.

This study described a difficult case with three unsuccessful attempts at removing the calcified stent by cystoscopy and extracorporeal lithotripsy. We finally opted for extended pyelolithotomy due to the limitations of percutaneous nephrolithotomy for this case. The patient had no postoperative complications.

Calcification of the double-J stent is a common complication with a high rate of patient morbidity, requiring further surgical procedures and a consequent increase in financial expenses [19]. To avoid potential complications with the use of a double-J stent, the use of a spreadsheet available on the Internet is a viable control option that could be fundamental to the management of patients so that they are not forgotten, the consequences of which are possible lawsuits [19–22].

The double-J stent is part of the urological arsenal for the treatment of urinary tract infections. Despite advances in recent years, the complications due to its presence in the ureter can be challenging to urologists and patients alike, leading to an increase in the morbidity rate and a financial burden on the healthcare system. Greater control of patients with stents should be exercised, counseling them on the maximum time of stent use and the complications that can arise in cases of forgetfulness.

## 5. Conclusion

Patients with complete coraliform calcification induced by a double-J ureteral stent should be treated in an individualized manner and considering the limitation of percutaneous surgery regarding the removal of all fragments.
